# Genome mining strategies for ribosomally synthesised and post-translationally modified peptides

**DOI:** 10.1016/j.csbj.2020.06.032

**Published:** 2020-06-25

**Authors:** Alicia H. Russell, Andrew W. Truman

**Affiliations:** Department of Molecular Microbiology, John Innes Centre, Norwich NR4 7UH, UK

**Keywords:** RiPP, Genome mining, Bioinformatics, Antibiotic, Natural product, Biosynthesis, BGC, biosynthetic gene cluster, NP, natural product, RiPP, Ribosomally synthesised and post-translationally modified peptide, PTM, post-translational modification, RTE, RiPP tailoring enzyme, ORF, open reading frame, HMM, hidden Markov model, DNN, deep neural network, MS, mass spectrometry

## Abstract

Genome mining is a computational method for the automatic detection and annotation of biosynthetic gene clusters (BGCs) from genomic data. This approach has been increasingly utilised in natural product (NP) discovery due to the large amount of sequencing data that is now available. Ribosomally synthesised and post-translationally modified peptides (RiPPs) are a class of structurally complex NP with diverse bioactivities. RiPPs have recently been shown to occupy a much larger expanse of genomic and chemical space than previously appreciated, indicating that annotation of RiPP BGCs in genomes may have been overlooked in the past. This review provides an overview of the genome mining tools that have been specifically developed to aid in the discovery of RiPP BGCs, which have been built from an increasing knowledgebase of RiPP structures and biosynthesis. Given these recent advances, the application of targeted genome mining has great potential to accelerate the discovery of important molecules such as antimicrobial and anticancer agents whilst increasing our understanding about how these compounds are biosynthesised in nature.

## Graphical abstract

**Figure:**
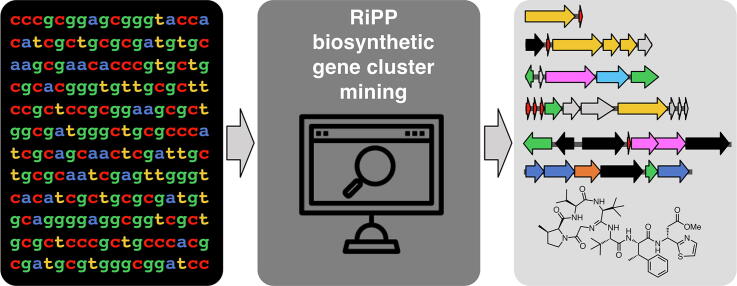

## 1 Introduction

Microorganisms and plants produce a plethora of natural products (NPs) with a range of bioactivities including antimicrobial, anticancer, pesticidal and immunosuppressive. As a result, many of these compounds are highly valuable and extensively utilised in medicine, agriculture and the food industry [1], [2]. In bacteria, the genes encoding NP biosynthetic pathways are typically clustered tightly together on the chromosome as biosynthetic gene clusters (BGCs). These genomic regions include genes for biosynthetic precursors, tailoring enzymes, regulation, transport and resistance elements [3]. Some bacteria have a particularly complex specialised metabolism, with actinomycetes such as *Streptomyces* species harbouring between 20 and 40 BGCs [3], [4], [5]. Fungal genomes also contain multiple specialised metabolite BGCs [6], [7], [8]. However, much of this microbial biosynthetic capacity is currently unexplored. Microbes only express limited numbers of their BGCs under laboratory conditions, and many microorganisms are uncultivatable [9], making the isolation of novel compounds challenging. In order to uncover the cryptic biosynthetic potential of microorganisms, genomics-based strategies have become powerful and increasingly popular methods for the automatic detection of biosynthetic genes [10]. Before the advent of genome mining, the identification of novel bioactive metabolites typically involved labour-intensive cultivation and screening of microbial extracts. As well as this being a highly time-consuming procedure, activity-based screening is also hindered by high rediscovery rates [11], [12], [13]. In contrast, a major challenge of genome mining is that it is difficult to predict which BGCs will produce molecules with desirable bioactivity, especially if a BGC is very different to previously characterised BGCs.

The first bacterial genome was sequenced in 1995, from *Haemophilus influenzae* [14]. Seven years later, the first *Streptomyces* genome was sequenced from *Streptomyces coelicolor* A3(2) [4], which provided the first evidence that actinomycetes contain many more BGCs for specialised metabolites than previously thought [3]. Since then, as sequencing technologies have become more advanced, accessible and cheaper, the number of prokaryotic genome sequences that are publicly available exceeds 200,000 (NCBI, March 2020). This wealth of genomic information has led to the development of multiple genome mining tools that survey this genomic data to automatically detect and annotate potential BGCs, typically by using algorithms that are based on knowledge of NP biosynthetic machinery. As well as identifying novel compounds from bacteria that are known to be talented producers of specialised metabolites, genome mining can also serve as a valuable tool to understand the biosynthetic potential of underexplored genera. Therefore, there is great potential for genome mining strategies to revitalise the antibiotic pipeline, at a time when discovery rates are dwindling and antimicrobial resistance is increasing [13].

## 2 Ribosomally synthesised and post-translationally modified peptides (RiPPs)

**Fig. 1:**
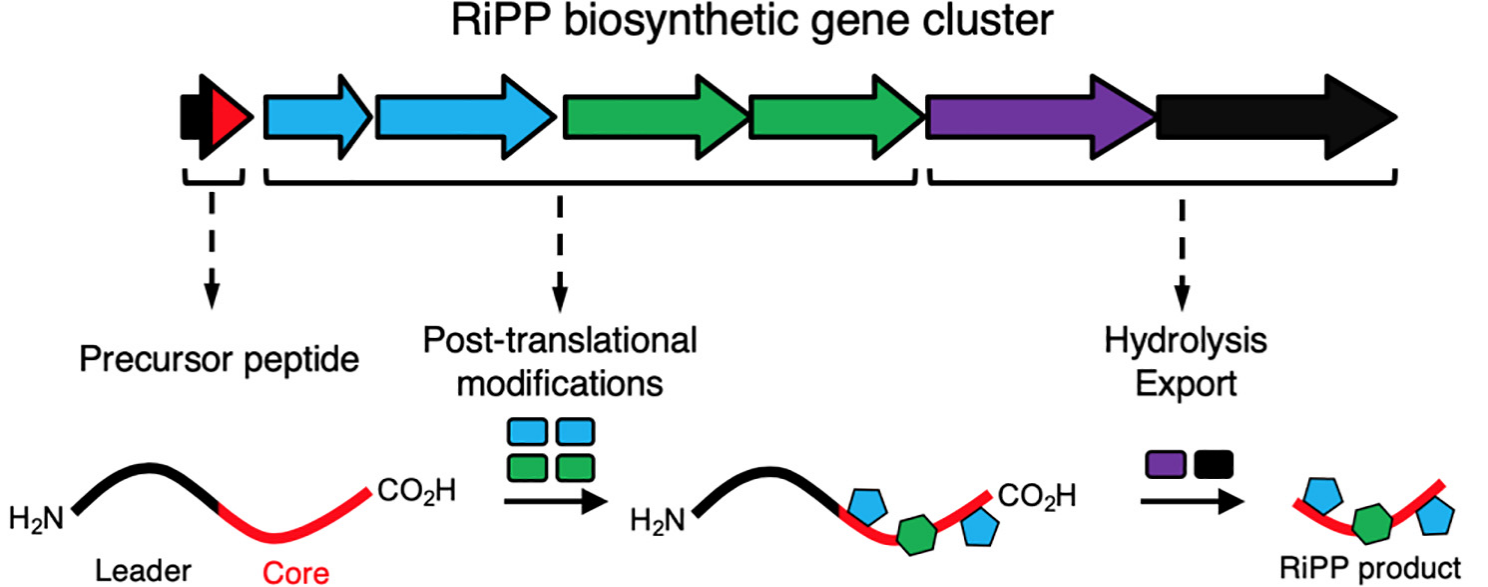
Schematic of RiPP biosynthesis.

RiPPs are a class of peptide NP harbouring post-translational modifications (PTMs) that give rise to a high degree of structural and chemical complexity [15]. RiPPs are produced from a short precursor peptide (PP) comprised of a leader peptide and a core peptide (Fig. 1). The PP is synthesised by the ribosome, and the core peptide is post-translationally modified by a series of RiPP tailoring enzymes (RTEs) that install various structural features onto the peptide backbone. The core peptide is usually cleaved from the leader peptide once most PTMs have been made, yielding a biologically active final product [15], [16] (Fig. 1). Leader peptides usually contain sequence motifs that act as recognition sequences for the RTEs to bind. A widespread mode of binding occurs via domains called RiPP precursor peptide recognition elements, which are present on many RTEs [17], [18], [19]. The leader peptide is also thought to play a protective role, preventing the core region from proteolytic cleavage before the biosynthetic post-translational modifications are complete [15]. In some examples, RiPPs contain a follower region instead of, or in addition to, the leader region [20].

**Fig. 2:**
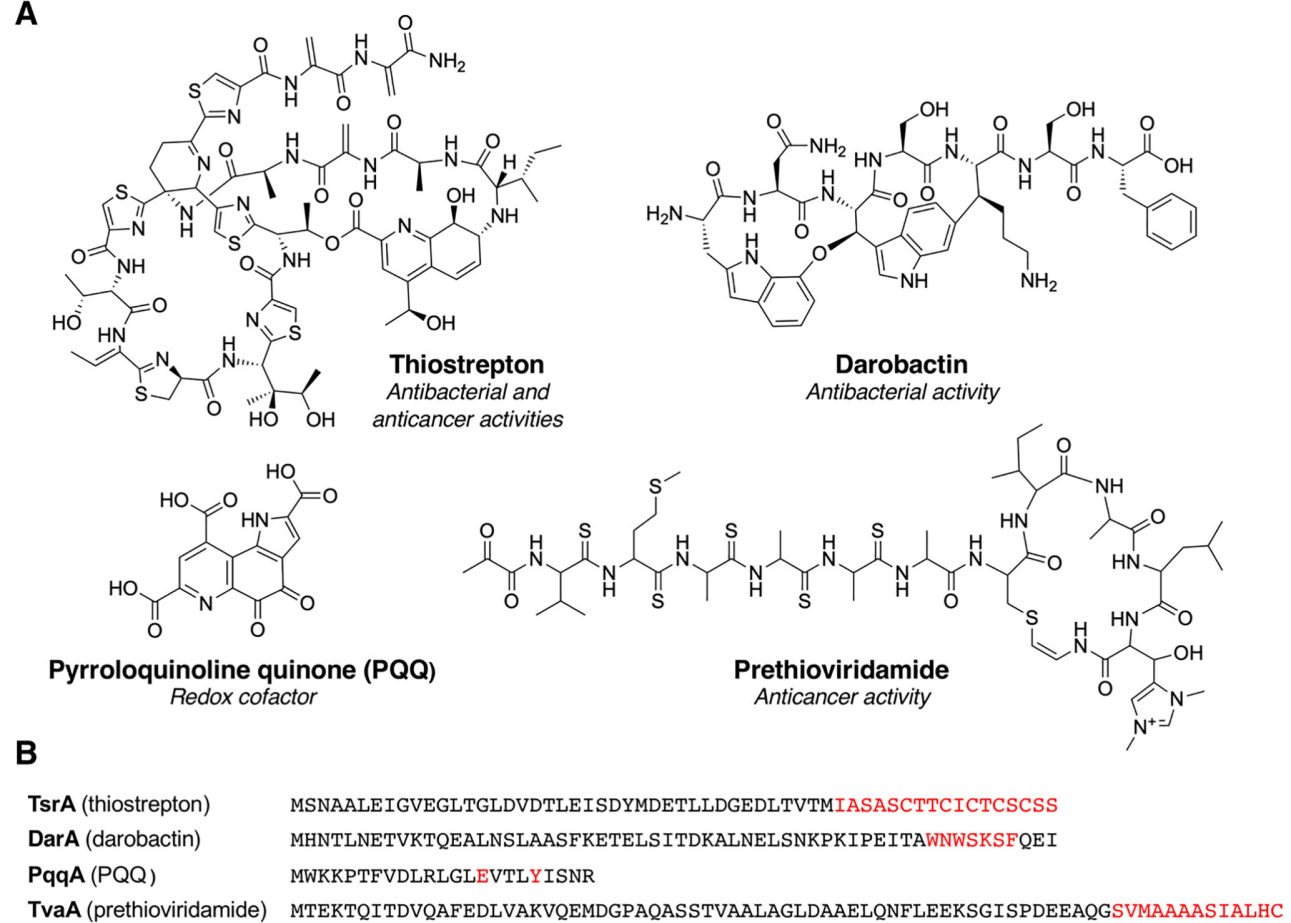
Examples of RiPP natural products. A. Structures of a thiopeptide (thiostrepton), a recently discovered antibiotic (darobactin), a redox cofactor (pyrroloquinoline quinone, PQQ) and a thioviridamide-like molecule (prethioviridamide). B. Precursor peptides corresponding to these RiPPs, where core peptides are coloured red.

The term ‘RiPP’ was only formally coined in 2013 [15], but characterised members of this NP class date back to as early as 1928, with the discovery of the lanthipeptide nisin [21], an antibacterial peptide that is still used as a food preservative today. RiPPs are grouped into multiple families based on their varied biosynthetic machinery and structural features. RiPP classes that have been characterised to date include linear azoline-containing peptides [22], [23], bottromycins [24], thiopeptides [25], [26], thioviridamide-like molecules [27], [28], lanthipeptides [29], [30], cyanobactins [31], [32], lasso peptides [33], [34], sactipeptides (peptides with sulfur-to-α carbon cross-links) [35], [36] and linaridins [37], [38]. These molecules display diverse bioactivities (Fig. 2A).

Unfortunately, genome mining for novel RiPPs presents several challenges. Unlike other classes of NP such as polyketides and nonribosomal peptides that are produced by multi-modular complexes, the biosynthetic logic of RiPPs means that there are few conserved features across the class, with RTEs varying between different RiPP families. Furthermore, RiPP PPs are very short (often < 50 amino acids, Fig. 2B) and are sometimes not annotated in genomes. Despite these challenges, genome mining for RiPPs presents an exciting opportunity to discover previously untapped biochemical diversity. Increased knowledge about RiPP biosynthetic mechanisms has allowed for improved algorithms for RiPP BGC detection. Improved knowledge of PP sequences can also aid in structural prediction and provide information on interactions between the PP and its cognate RTEs. The use of targeted genome mining therefore represents a powerful strategy to accelerate future RiPP discovery.

## 3 RiPP genome mining tools

General reviews of microbial genome mining have previously been published [10], [39], [40], [41], [42], [43], [44], but this review will focus on the plethora of genome mining tools that have been specifically developed for RiPPs in recent years. While there are similarities amongst a number of these tools, they each have different strengths and there are substantial differences in how some tools operate. Tools like antiSMASH [45], [46], [47], [48], [49] function by analysis of a single genome (and integrate the RiPP output with analysis of other BGC classes), whereas others such as RODEO [36], [50] and RiPPER [51] function optimally in a pan-genome mode and enable the user to define RTEs. A number of these tools provide additional outputs, including the prediction of PP sequences, leader peptide cleavage sites, PTMs and final product structures, as well as associating sequence data with mass spectrometry data. All of these tools are summarised in Table 1, Table 2 and are described below in order of when they were first described. Online databases containing information about known RiPP molecules and BGCs are also reported (Table 3). Finally, we carry out a comparative analysis of selected tools that assess the biosynthetic potential of whole genomes.

**Table 1:** Summary of genome mining tools available for RiPPs.

**Tool**	**Web address**	**Function and RiPP class**	**Interface**	**Input**	**Output**
BAGEL4	http://bagel4.molgenrug.nl/	• BGC identification and annotation • Multiple RiPP classes	Web	Sequence file (FASTA) or built-in set of publicly available genomes in RefSeq database	• Html output showing BGC regions with gene annotations • Sequence alignment with curated precursor peptides • Downloadable GenBank files, FASTA files, gene tables and promoter/terminator information
antiSMASH5	https://antismash.secondarymetabolites.org/	• BGC identification, annotation and analysis • Multiple RiPP classes	Web	Sequence file (FASTA, GenBank or EMBL) or NCBI nucleotide accession	• Html output showing BGC regions with gene annotations and predicted class • Predicted PP and cleavage sites for some RiPP classes • Downloadable GenBank files and other data for BGC regions • KnownClusterBlast analysis
PRISM4	http://grid.adapsyn.com/prism/#!/prism	• BGC identification, PP cleavage and PTM prediction • Multiple RiPP classes	Web	Sequence file (FASTA or GenBank)	• Html output showing BGC regions with gene annotations and predicted class • Predictions of core peptide and final structures • SMILES strings for predicted structures, FASTA sequences of BGCs
RiPPMiner	http://202.54.226.242/~priyesh/rippminer2/new_predictions/index.php	• BGC identification and RiPP class • Predictions of structure, cleavage and crosslinks • Multiple RiPP classes	Web	Peptide = PP sequence (raw or FASTA)	*Peptide* • Html output with predicted structure and class • SMILES strings for predicted structures
				Genome = sequence file (FASTA)	*Genome* • Html output showing identified clusters and annotations as well as peptide cleavage, crosslinks and structural predictions • SMILES strings of predicted structures • List of other small ORFs present in BGC
RODEO2	http://ripp.rodeo/index.html	• RiPP BGC identification, PP identification and structural prediction • Lasso peptides, lanthipeptides, thiopeptides & sactipeptides	Web or Python	List of bait protein accession numbers. Optional: HMMs and configuration file	• Html files with BGC information and Pfam domain annotation • .csv files of PP sequences and BGC Pfam domains
RiPPER	https://github.com/streptomyces/ripper	• PP and BGC recognition • Class independent	Docker	List of bait protein accession numbers	• GenBank files of retrieved BGCs annotated with short peptides • Table of PP data • RODEO files for retrieved BGCs
NeuRiPP	https://github.com/emzodls/neuripp	• PP recognition • Class independent	Python	PP sequence file (FASTA)	• File of sequences classified by NeuRiPP as positive PPs • Separate file of non-RiPP peptides

**Table 2:** Summary of MS-based mining tools available for RiPPs.

**Tool**	**Web address**	***Function and RiPP class***	**Interface**	**Input**	**Output**
RiPPquest/MetaMiner	https://github.com/ablab/npdtools	• MS-guided genome mining, optimised for large datasets • Multiple RiPP classes	Python or web (GNPS)	LC-MS/MS data file (MGF, mzXML, mxML or mzData) and sequence file (FASTA, antiSMASH GenBank output or BOA txt output)	• .tsv files with information about identified peptides and RiPP class
	http://gnps.ucsd.edu/ProteoSAFe/static/gnps-theoretical.jsp				
Pep2Path	http://pep2path.sourceforge.net/	• BGC identification from peptide MS data	Python	Comma-separated sequence of mass shifts or amino acids, and a sequence file (FASTA, GenBank or EMBL)	• Table with best peptide matches
CycloNovo	https://github.com/bbehsaz/cyclonovo	• Cyclopeptide identification and prediction	Python or web (GNPS)	MS data file (mzXML or MGF)	• MGF file of identified cyclopeptide spectra • Spectra listed with cyclopeptide scoring (txt) • Peptide sequencing reconstructions (txt)
	https://gnps.ucsd.edu/ProteoSAFe/index.jsp?params=%7B%22workflow%22:%22CYCLONOVO%22%7D				
DeepRiPP	http://deepripp.magarveylab.ca/	• PP structural and class predictions • BGC identification • Multiple RiPP classes	Web	NLPPrecursor: PP sequence (FASTA)	• NLPPrecursor: Html output of predicted RiPP class and cleavage site
				BARLEY: core peptide sequence and RTE	• BARLEY: Html output of alignment with similar RiPPs and structure predictions
				CLAMS: MS data (mzML)	• CLAMS: Html output with list of MS peaks
				DeepRiPP (full): sequence file (FASTA) and optional MS file (mzML)	• DeepRiPP (full): integrated Html output of NLPPrecursor, BARLEY and CLAMS • Attempted matching between structure prediction and MS data

**Table 3:** Summary of databases available for RiPPs and their BGCs.

**Database**	**Link**	**Features**
ThioBase	https://db-mml.sjtu.edu.cn/THIOBASE/	• Thiopeptide specific • Structure and activity • BGCs and core peptide sequences • Literature links
BACTIBASE	http://bactibase.hammamilab.org/main.php	• Structural and physiochemical properties of bacteriocins • Literature and sequence database links
BAGEL database	http://bagel4.molgenrug.nl/databases.php	• RiPP and bacteriocins • Precursor peptide sequences • Literature and sequence database links
RiPPMiner database	http://www.nii.ac.in/~priyesh/lantipepDB/new_predictions/index.php#/~priyesh/lantipepDB/new_predictions/second.php	• RiPP structures • Precursor peptide sequences and modified residue details • Literature links
IMG-ABC	https://img.jgi.doe.gov/cgi-bin/abc-public/main.cgi	• NP BGC database from all genomes in IMG • All antiSMASH-identified NP classes • Searchable by BGC class
MIBiG	https://mibig.secondarymetabolites.org/	• Repository of NP BGCs • Searchable by BGC class • Structure and BGC details • Literature links
antiSMASH database	https://antismash-db.secondarymetabolites.org	• antiSMASH outputs for sequenced bacterial genomes • All antiSMASH-identified NP classes • Searchable by BGC class

### 3.1 BAGEL

BAGEL (BActeriocin GEnome mining tooL) is one of the earliest tools developed for the identification of RiPP and bacteriocin BGCs. First released in 2006, it was built to address the issue that open reading frames (ORFs) with limited sequence homology are difficult to annotate [52]. BAGEL searches for RiPPs (also defined as class I bacteriocins by BAGEL), class II bacteriocins (small heat stable proteins < 10 kDa) and class III bacteriocins (large heat-labile proteins > 10 kDa). BAGEL identifies putative RiPP and bacteriocin ORFs using knowledge-based peptide and motif databases, combined with information about the genetic context of accessory genes for processing, modification, transport and regulation of RiPPs and bacteriocins. Initial screening identifies areas of interest in which ORFs are identified. Small ORFs are subsequently searched for in the intergenic regions and are analysed by BLAST against curated databases for each type of bacteriocin described above. If homology is found, an alignment is produced along with predictions of promoters and terminators [52].

Since its first release, updates to the software have provided further optimisation of RiPP detection. BAGEL2 implemented extended use of profile hidden Markov models (HMMs) and updated the manually curated databases of known bacteriocins and accessory genes in order to incorporate improved biosynthetic knowledge. An advanced classification algorithm was also implemented to allow prediction of subclasses of bacteriocins [53]. BAGEL3 included implementation of new HMM models for tailoring genes involved in the biosynthesis of cyanobactins, sactipeptides and linaridins [54]. The most recent update to the software is BAGEL4, whose annotation database was updated with improved RiPP protein domain information [55]. As well as BGC identification, the BAGEL web server also provides a peptide database containing information about almost 500 RiPPs and bacteriocins (Table 3). The BAGEL4 “Core Peptide Blast” function enables the user to search against this database using a user-defined set of precursor peptide sequences.

### 3.2 antiSMASH

antiSMASH (antibiotics and Secondary Metabolite Analysis Shell) is a genome mining tool for the identification and analysis of 52 types of NP BGC. It was first released in 2011 [45] and has been updated several times [46], [47], [48], [49]. antiSMASH is the most widely used genome mining tool, with over 670,000 jobs processed online at the time of writing. As well as bacterial genome mining, antiSMASH also has platforms that are optimised for fungal (fungiSMASH) [48] and plant (plantiSMASH) [56] genomes. antiSMASH works by comparing encoded gene products with a manually curated library of HMMs, which describe a range of NP biosynthetic genes. BGCs are identified by assigning key enzymes present in a gene cluster to specialised metabolite-specific clusters of orthologous groups. Further downstream analyses are also carried out to annotate accessory genes, predict BGC boundaries, and to predict final structures of compounds. The integrated KnownClusterBlast feature enables the comparison of identified BGCs with known BGCs present in the MIBiG database [57] (Table 3) [45], [46].

Although not solely focused on RiPPs, successive updates to antiSMASH have incorporated numerous features that provide a detailed RiPP BGC annotation. antiSMASH 2.0 added support for thiopeptide and sactipeptide BGC recognition [46] and antiSMASH 3.0 included improved analysis of lanthipeptide structures and modifications, as well as integration with ClusterFinder [58], which is a HMM-based algorithm that identifies BGCs based on the co-occurrence of Pfam domains associated with biosynthesis. antiSMASH 3.0 also included the newly adopted RiPP nomenclature that was published in 2013 [15]. antiSMASH 4.0 incorporated the RODEO [50] algorithm (see below) to help evaluate candidate PPs for lasso peptides, thiopeptides, class I lanthipeptides and sactipeptides [48]. In its current release, antiSMASH 5.0 includes refined rules for lanthipeptides, linear azoline-containing peptides, radical SAM-associated RiPPs and fungal RiPPs [49]. Overall, antiSMASH harbours algorithms to detect a range of different RiPP families, and has been successfully used to guide the discovery of novel RiPPs, such as the lanthipeptide streptocollin [59].

### 3.3 ThioFinder

ThioFinder, released in 2012 [60], was specifically developed to identify thiopeptide BGCs. ThioFinder requires a nucleotide sequence as an input and uses this to search for conserved thiopeptide biosynthetic genes such as YcaO-domain proteins and lanthipeptide-like dehydratases. These searches are based on HMMs. PPs within a candidate thiopeptide BGC are then identified by searching for characteristic motifs, such as ‘SCTT[CS][GI]CT[CS]S[CS]’, which was identified through a MEME analysis [61] of known thiopeptide PPs. This allows for subsequent detection and annotation of corresponding thiopeptide BGCs. ThioFinder was used to identify 54 new thiopeptide BGCs and grouped these into three types, thus revealing previously untapped thiopeptide diversity.

### 3.4 RiPP-PRISM

**Fig. 3:**
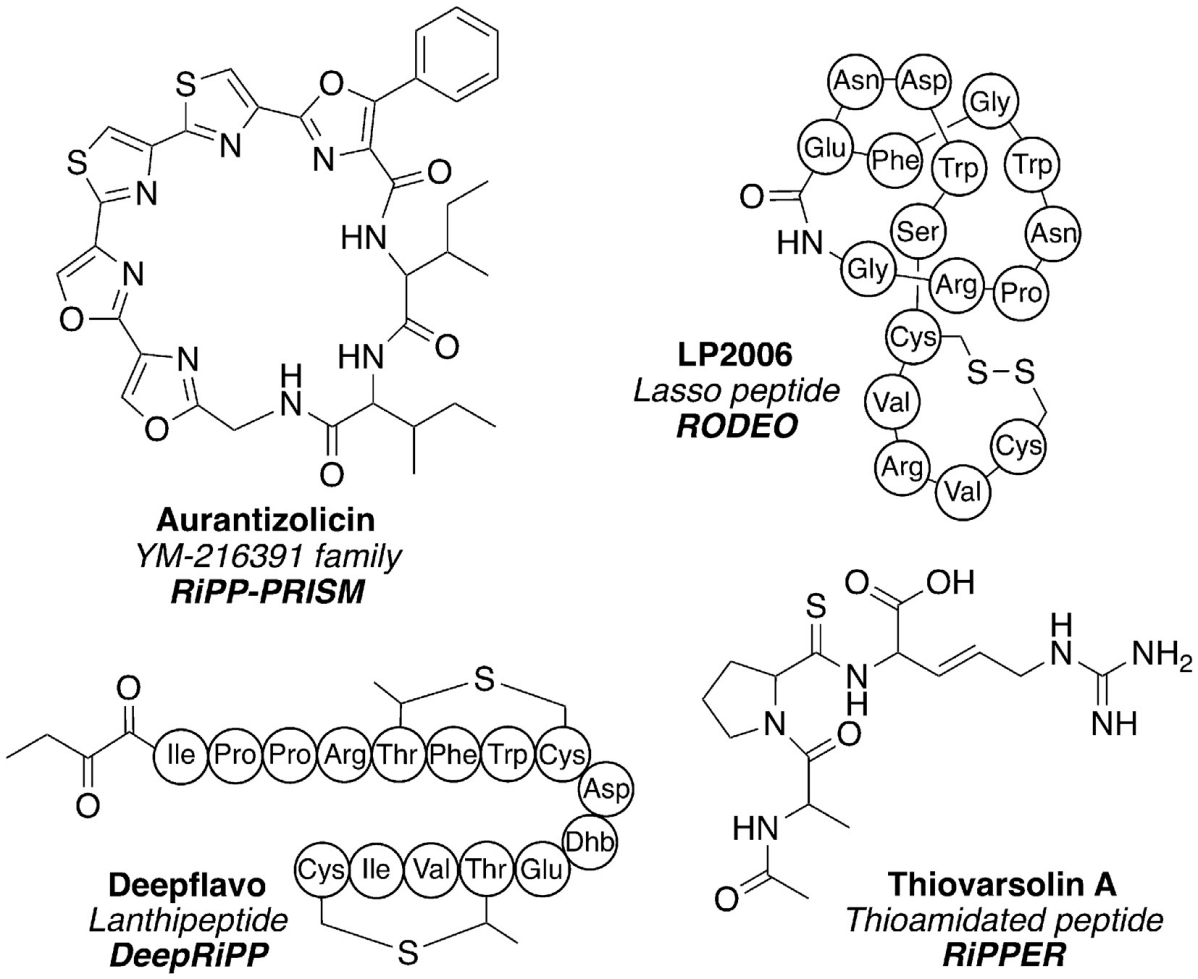
Examples of RiPPs whose discovery was guided by the use of genome mining tools. The compound name, class and tool are listed alongside each structure.

RiPP-PRISM, released in 2016, is a tool that identifies BGCs and predicts structures for 21 families of RiPPs [62]. It integrates into the previously developed PRISM [63], [64] (PRediction Informatics for Secondary Metabolomes) tool, a platform for the identification of non-ribosomal peptide and polyketide BGCs and associated structures. PRISM was extended to cover RiPPs by building libraries of 58 motifs, 154 HMMs and 94 virtual PTMs specific to RiPP biosynthesis. This knowledge is used to predict PP cleavage and final structures. RiPP-PRISM was used to investigate the chemical space occupied by RiPPs by analysing the 65,421 prokaryotic genomes listed in NCBI at the time, leading to the identification of over 30,000 RiPP BGCs. RiPP-PRISM analysis suggested that 82% of genetically encoded RiPPs remain unknown, but this figure is likely to be an underestimation given that numerous recently discovered RiPP families are not covered by RiPP-PRISM. As well as identifying previously unknown BGCs, RiPP-PRISM was also used to facilitate targeted identification of novel RiPPs, leading to the isolation of aurantizolicin from *Streptomyces aurantiacus*, a cyclic azoline-containing compound closely related to YM-216391 [62] (Fig. 3).

### 3.5 RiPPMiner

RiPPMiner, released in 2017, is a bioinformatics resource for predicting chemical structures and classes of RiPPs, as well as identifying novel BGCs [65]. The aim of RiPPMiner is to predict complex chemical structures from the precursor peptides of selected classes of RiPP, including lanthipeptides, lasso peptides, cyanobactins and thiopeptides. This uses support vector machine and random-forest classifiers trained on over 500 experimentally characterised RiPPs, which are used to distinguish RiPP PPs from other small proteins and classify identified precursors into 13 RiPP families. Unlike tools such as RiPP-PRISM and antiSMASH that use HMMs of RTEs to predict the RiPP class, RiPPMiner uses a machine learning model trained using the amino acid sequence of the RiPP PP gene alone to identify RiPPs and then predict their class.

RiPPMiner includes two different modules: peptide and genome. RiPPMiner-peptide takes a PP sequence and provides predictions about class, structure, crosslinks and cleavage sites for selected RiPP families, such as lanthipeptide, cyanobactin, lasso peptide and thiopeptide. RiPPMiner-genome predicts chemical structures and identifies BGCs from a genomic sequence. Like BAGEL and ThioBase, RiPPMiner also includes a publicly available database of known RiPPs, RiPPDB, containing information about structures and biosynthetic genes (Table 3).

### 3.6 RODEO

RODEO (Rapid ORF Description and Evaluation Online), released in 2017, is a tool developed for the analysis of RiPP BGCs and prediction of PP sequences and structures [50]. Unlike previously described genome mining tools that analyse a whole genome or precursor peptide sequence, RODEO uses a protein of interest as input and captures the surrounding genomic environment to identify nearby biosynthetic genes, and thus new BGCs. The RODEO algorithm combines HMM-based analysis, heuristic scoring, motif analysis and machine learning to identify precursor peptides and predict cleavage sites between leader and core peptides. RODEO was first used to survey and annotate the genomic space occupied by lasso peptides, revealing over 1,400 lasso peptide BGCs. Previously uncharacterised tailoring enzymes were also observed. Several new lasso peptides were characterised following RODEO analysis, including LP2006 from *Nocardiopsis alba*, which forms a novel ‘handcuff’ topology (Fig. 3), and citrulassin A from *Streptomyces albulus*, which bears a unique RiPP PTM where an Arg residue is modified to citrulline [50].

The RODEO algorithm was further developed for the analysis of thiopeptides. This was guided by the generation of a custom pHMM for the [4 + 2]-cycloaddition enzymes that generate a six-membered N-heterocycle in thiopeptides. This expanded the class by a factor of four and revealed multiple novel thiopeptide BGCs. A novel antibacterial thiopeptide called saalfelduracin was isolated from *Amycolatopsis saalfeldensis* [66]. RODEO2 has since been updated for the detection of class I lanthipeptides and sactipeptides, and was utilised to discover huazacin, a sactipeptide with activity against *Listeria monocytogenes* [36]. This analysis identified further diversity in BGCs predicted to make sactipeptides, but also led to the experimental characterisation of a new RiPP family, the ranthipeptides (radical non-α thioether peptides).

### 3.7 RiPPER

RiPPER (RiPP Precursor Peptide Enhanced Recognition), released in 2019, is a tool for the discovery of novel RiPP PPs and associated BGCs. Like RODEO, RiPPER takes a putative RTE as an input, and identifies putative PPs in the surrounding genomic region. RiPPER was developed to overcome the limitation that many RiPP mining tools are restricted to discovery of specific RiPP families, and might therefore overlook untapped biochemical novelty [51]. RiPPER uses the RODEO2 [66] script to capture genomic regions centred on the ‘bait’ RTE, and a modified version of Prodigal [67], Prodigal-short, is employed to reannotate the captured genetic region for likely protein-coding sequences that could be RiPP PPs. The peptides with the highest Prodigal-short scores are retrieved and assessed for numerous characteristics, including conserved domains, such as Pfam domains and RiPP-specific HMMs from NCBI. Subsequent networking analysis of identified PPs with EGN [68] is used to help identify families of related PPs. RiPPER is therefore best suited to the analysis of multiple related BGCs, and was shown to successfully identify families of precursor peptides for lasso peptides, thiopeptides and microviridins without any prior knowledge of precursor peptide sequence motifs.

RiPPER was used to assess the unexplored diversity of thioamidated RiPPs using an input of TfuA-like proteins from Actinobacteria. 743 peptides were retrieved which grouped into 74 distinct networks of peptides. Analysis of one of these networks led to the characterisation of the thiovarsolins from *Streptomyces varsoviensis*, a new structural class of thioamidated RiPP (Fig. 3). Due to the input of user-defined protein accessions as a starting point for analysis, RiPPER is a flexible tool that can be applied to various RiPP classes [51] and can be used to identify precursor peptides that have no homology to known families of RiPP, as well as short peptides that contain RiPP PP domains. It also provides an accurate reannotation of genomic loci for small genes missed by automated genome annotations.

### 3.8 NeuRiPP

NeuRiPP [69], released in 2019, is a tool for RiPP PP identification that does not require genomic context. The premise of NeuRiPP was to build a tool that could discriminate genuine PPs from false positives in a given list of sequences, thereby overcoming the challenge when some genome mining approaches such as RiPPER [51] might identify large numbers of peptide coding sequences. NeuRiPP is built from a PP dataset that was used to train a deep neural network (DNN). The positive dataset was constructed by collating experimentally validated PPs as well as sequences from PRISM [63], ThioFinder [60], RODEO [50], RiPPER [51] and antiSMASH [49]. The negative dataset was made from peptides shown not to be genuine RiPP precursors. The neural network was thus trained on over 9,454 sequences. The DNN was then used to classify short peptides on their likelihood of being genuine RiPP PPs, with the best network architecture achieving over 99% accuracy. NeuRiPP was able to identify the novel thioamidated peptides identified previously by RiPPER [51] and also complemented predictions made by RODEO [50]. As well as identifying PPs enriched with HMMs for known RiPP precursors, NeuRiPP was also able to successfully identify putative precursors for RiPP classes it was not trained on. The flexibility of neural networks allows for future improvements, as more PPs can be added to the training dataset as they are discovered. NeuRiPP is therefore a promising tool for RiPP discovery that starts with PPs instead of biosynthetic enzymes for the identification of BGCs.

### 3.9 Bespoke approaches

A 2011 review by Velasquez and van der Donk summarises the foundational approaches used to mine for new RiPP BGCs [70], such as the identification of the lasso peptide capistruin from *Burkholderia thailandensis* E264 [71]. An early systematic approach at identifying lasso peptide BGCs was reported by Link and colleagues in 2012 [72], who developed a pattern matching algorithm using conserved amino acids in lasso PPs. This was used to direct the discovery of astexin-1.

Despite the development of the genome mining tools described in this review, the diversity of RiPP BGCs still necessitates bespoke approaches for the discovery of novel RiPPs that do not conform to the bioinformatic rules used by these tools. Haft has used computational approaches to predict multiple novel RiPP BGC families, including mycofactocin, a RiPP predicted to be widespread in mycobacteria [73]. To identify the mycofactocin BGC, partial phylogenetic profiling was used to identify conserved genomic loci associated with genes encoding a clade of radical SAM proteins. The BGCs were then reannotated to identify a conserved yet previously unannotated mycofactocin PP gene. Subsequent experimental studies have proven this to be a genuine RiPP pathway [74], [75]. Haft and Mitchell bioinformatically identified Nif11-like and nitrile hydratase-like leader peptides associated with BGCs that had homology to linear-azoline containing peptide BGCs [76]. Subsequent studies from the Piel group have experimentally characterised new RiPP families that derive from peptides with nitrile hydratase-like leader peptides, including peptides with extensive D-amino acids that are introduced by radical SAM epimerases [77]. An alternative approach by the Seyedsayamdost group searched for quorum sensing-regulated, radical SAM enzyme-containing BGCs, leading to the identification of around 600 novel RiPP BGCs. One subclass of these RiPPs harboured a unique PTM, in which four unactivated positions in the side-chains of Trp and Lys are linked by two C–C bonds to form a substituted tetrahydro [5-6]benzindole moiety, a reaction carried out by a single radical SAM enzyme [78].

## 4 Mass spectrometry-guided genome mining

Mass spectrometry (MS) is a powerful technique that is widely used in NP research [79]. MS approaches have also been integrated into several genome mining tools. The launch of Global Natural Products Social (GNPS, https://gnps.ucsd.edu/ProteoSAFe/static/gnps-splash.jsp) molecular networking has massively benefitted NP discovery [80]. This uses tandem MS (MS/MS) to identify families of related compounds in spectra the user uploads, and compares this to a large database of MS/MS spectra. This opened up the potential to utilise a vast amount of publicly available metabolomic datasets for NP discovery. Analysis of metabolomic data can be useful in the context of peptidic NPs such as RiPPs, as fragmentation patterns can provide key information about the identity and order of amino acid residues present in molecules, as well as post-translational modifications that correspond to characteristic mass losses. However, extensive post-translational modifications also provide a substantial challenge for automating RiPP identification using MS-based methods, given that modifications can affect fragmentation patterns and MS/MS mass losses. This contrasts with conventional MS/MS-based proteomics.

One of the first examples of MS-guided genome mining was demonstrated in 2011 by Kersten *et al.* with Natural Product Peptidogenomics (NPP) [81]. NPP was developed in order to help connect chemotypes of peptide NPs such as RiPPs to their BGCs. NPP took advantage of the recent technological advances in MS and genomics, as well as knowledge of peptide NP biosynthesis. The NPP workflow starts with MALDI-TOF MS analysis and searches for masses between 1,500–5,000 Da. Putative peptides are then identified based on MS^n^ fragmentation patterns, which are used to generate peptide sequence “search tags” that are compared to the six-frame translation of the genome to identify candidate precursor peptides. Knowledge of RiPP biosynthetic logic is implemented, and the NPP workflow includes several iteration steps that ensure that a match of peptide MS^n^ data to a genomics-derived peptide structure makes sense biosynthetically. With this approach, NPP was able to identify several examples of previously unidentified RiPPs including lanthipeptides, lasso peptides and linaridins from a range of *Streptomyces* strains. Since the development of NPP, a number of publicly available tools are now available that use MS and genomic data to guide RiPP discovery. These are summarised below and in Table 2.

### 4.1 RiPPquest and MetaMiner

RiPPquest was developed as a combined metabolomic and genome-guided mining tool for the identification of microbial RiPPs [82], specifically lanthipeptides, with the aim of overcoming limitations of previous MS-based tools. For example, the sequence tagging method of NPP may lead to macrocyclic RiPPs being missed, as the long sequence search tags are often not present. When RiPPquest was released in 2014 it was the first genome mining tool to automate both BGC prediction and connection with MS/MS data. The RiPPquest workflow starts with the prediction of lanthipeptide BGCs and putative PPs from a target microbial genome. MS/MS spectra of all possible final lanthipeptide structures are then calculated for each putative core peptide based on all possible PTMs. Next, the peptide-spectrum matches are scored in order to identify connections between metabolomic and genomic data. Finally, a molecular network is generated from the MS/MS data set, in order to identify homologues of characterised lanthipeptides and families of related peptides from top-scoring peptide-spectrum matches. RiPPquest was successfully used to characterise a new class II lanthipeptide called informatipeptin from *Streptomyces viridochromogenes.*

Despite this success, RiPPquest was limited to the discovery of lanthipeptides from small datasets and could only search for a predefined set of PTMs. To address these limitations, the same research teams released MetaMiner as a replacement in 2019 [83], which is designed to search for lanthipeptides, linear azoline-containing peptides, lasso peptides, linaridins, glycocins, cyanobactins, proteusins, phenol-soluble modulins and auto-inducing peptides. MetaMiner is integrated into GNPS (http://gnps.ucsd.edu/ProteoSAFe/static/gnps-theoretical.jsp) and is also available as part of the Natural Product Discovery tools package (https://github.com/ablab/npdtools). MetaMiner works by first analysing the paired genome/metagenome assemblies and MS/MS data from a given sample set. From this (meta)genomic data, MetaMiner identifies putative BGCs and corresponding PPs using antiSMASH and Bacteriocin Operon and gene block Associator [84], and then constructs target and decoy putative RiPP structure databases. Here, it can either function in a fast “motif-ORF” (RiPP motif finding) or a slower “all-ORF” (genome six-frame translation) to search for putative PPs. Benchmark testing of these modes highlighted that each mode has its own advantages in terms of statistically significant PP detection. Notably, motif-ORF will miss PPs with novel motifs, but typically provides better statistical significance to predictions. Tandem mass spectra are then compared against these databases, and the set of described RiPPs is expanded via mass spectral networking. The decoy database is formed from randomly shuffled ORFs and is used to estimate false discovery rate.

The application of MetaMiner led to the identification of 31 known and seven unknown RiPPs in datasets from multiple bacterial taxa including *Actinomyces*, *Bacillus* and Cyanobacteria, as well as numerous microbial sources, such as a sponge microbiome, the International Space Station and the human microbiome.

### 4.2 Pep2Path

At a similar time to the release of RiPPquest, Medema *et al* released Pep2Path, a tool for MS-guided genome mining of peptide NPs [85]. Two algorithms were implemented to achieve this: one for non-ribosomally synthesised peptides (NRP2Path) and one for RiPPs (RiPP2Path). To match RiPP molecules to their PPs, RiPP2Path converts a series of MS/MS mass shifts into possible amino acid sequences to generate search tags. It then attempts to match these tags to the six translation frames retrieved from (meta)genomic sequences. Unlike RiPPquest, RiPP2Path was designed to identify PPs of any type, although there is limited information on how well it handles heavily modified RiPPs. RiPP2Path is unlike other MS-based tools in that the required input comprises mass shift or amino acid sequences rather than raw MS data.

### 4.3 Hypothetical Structure Enumeration and Evaluation (HSEE)

Released in 2014, the goal of HSEE was to predict the structure of an unknown RiPP using a combination of the accurate molecular weight, tandem MS data and the types of PTMs predicted from the genetic or biochemical information (R scripts available in supplementary information of the HSEE paper) [86]. HSEE is designed to aid in the structural elucidation of RiPPs where MS/MS data and a BGC is available. This does require the user to input possible mass changes based on prior knowledge of likely post-translational modifications. Therefore, the tool is not designed to identify new RiPPs from complex datasets, but does allow the user to analyse multiple MS/MS spectra with different experimental settings in parallel, and thereby generate hypothetical structure scores to help characterise the associated RiPP. HSEE was used to determine the structure of prochlorosin 1.2, a lanthipeptide whose structure was not known.

### 4.4 CycloNovo

CycloNovo, released in 2020, is a tool for the detection of cyclic peptides including cyclic RiPPs [87]. This is available via Github or integrated into GNPS. Previous cyclic peptide detection algorithms have not been optimised for large mass spectral datasets and are limited to the discovery of known cyclic peptides and related variants [88], [89], [90]. In contrast to linear peptides, cyclic peptides provide a major challenge for MS/MS prediction, as they can theoretically fragment at any amide in the cyclic backbone, which provides a much more complex series of ions than linear peptides, as the resulting fragment will not necessarily match the primary amino acid sequence. CycloNovo overcomes this limitation by using de Bruijn graph representations of spectra. Here, putative k-mers (strings of k consecutive amino acids) are calculated for putative cyclopeptides and CycloNovo then scores these against input spectra [87]. de Bruijn graphs are used widely in DNA sequence assemblers but had not previously been applied to cyclic peptide sequencing.

CycloNovo first uses an algorithm to identify putative cyclic peptides in tandem MS datasets. CycloNovo then generates all combinations of predicted amino acids that have a total mass equal to the precursor mass and predicts k-mers for each combination (effectively the calculated MS/MS spectra for each putative peptide string within the cyclic peptide). These k-mers are defined as high-scoring if they match the spectrum. A de Bruijn graph is then constructed using these high-scoring k-mers. All feasible cycles are found in the de Bruijn graph that correspond to a peptide with the correct precursor mass and have a length equal to the number of predicted amino acids. These are then scored against the experimental spectrum to provide a p-value associated with the prediction. In contrast to other MS-based mining methodologies, this approach does not require or use any matching genomic data. Cyclospectra that are identified by CycloNovo from a given input file can be further analysed through GNPS to provide annotation using Dereplicator/Varquest [91], [92], or to identify molecular networks. CycloNovo was applied to GNPS datasets and found over 400 cyclic peptides that were previously unreported. In comparison, database search tools were only able to identify 81 known cyclopeptides. CycloNovo was also used to analyse a human stool dataset, which found several bioactive cyclopeptides from consumed food that had remained stable throughout the gastrointestinal system [87].

### 4.5 DeepRiPP

Building on the genomic and MS approaches described above, DeepRiPP (released in 2020) is an example of a tool that combines both genomic and metabolomic information to automate detection of RiPPs and their associated BGCs [93]. DeepRiPP is a three-stage modular platform, where users can either run analyses on individual steps or utilise the full DeepRiPP workflow. The first step involves a deep neural network-based tool, NLPPrecursor, which identifies PPs independent from their genomic context. This predicts the RiPP class for a given PP sequence and also predicts a cleavage site for the core peptide. The second step compares biosynthetic loci to known RiPP pathways using the Basic Alignment of Ribosomal Encoded Products Locally (BARLEY) algorithm. This infers RiPP biosynthetic reactions within the BGC and compares the predicted RiPP product with a database of characterised RiPPs. This provides a similarity score between the candidate BGC and known RiPPs, with the aim of prioritising RiPP novelty. The final step of DeepRiPP, Computational Library for Analysis of Mass Spectra (CLAMS), employs an algorithm that compares mass spectral data with identified RiPP BGCs. This involves matching the exact mass of a predicted RiPP and assessing for the presence of supporting MS/MS fragmentation patterns. DeepRiPP was capable of discriminating true RiPP precursors from non-RiPP ORFs, with a positive predictive value of 98% on a training set of RiPPs identified from RiPP-PRISM [62]. DeepRiPP was used to analyse 65,421 bacterial sequences where it identified 19,498 novel RiPPs. This analysis guided the identification of novel compounds, including deepstreptin, a lasso peptide, and two lanthipeptides, deepflavo (Fig. 3) and deepginsen [93]. Much like BAGEL and antiSMASH, DeepRiPP is mainly limited to the identification of representatives of known RiPP families.

## 5 RiPP databases

Numerous databases for RiPPs have been developed, providing information about sequences, structures and producers of known RiPP molecules (Table 3). More generally, databases for NP gene clusters have also been developed that include RiPP BGCs. antiSMASH has its own searchable database of BGCs from over 24,000 genomes [94], and is also associated with a number of other databases, including MIBiG (Minimum Information about a Biosynthetic Gene Cluster) and IMG-ABC (Integrated Microbial Genomes Atlas of Biosynthetic gene Clusters) [95]. IMG-ABC is a repository for known and predicted NP BGCs, containing information on over 400,000 BGCs including those for RiPPs, and also includes various search and analysis tools for genes and pathways. MIBiG is another repository of NP BGCs [57] that defines a community-approved set of information to describe BGCs. MIBiG provides information about biosynthetic genes, their products, class and producing organisms, and is used for the KnownClusterBlast feature of antiSMASH. In terms of RiPP-specific databases, the genome mining tools BAGEL, ThioFinder and RiPPMiner all feature associated databases, while BACTIBASE is a searchable database of lanthipeptides and class II/III bacteriocins.

## 6 Comparative analysis of genome mining tools

**Fig. 4:**
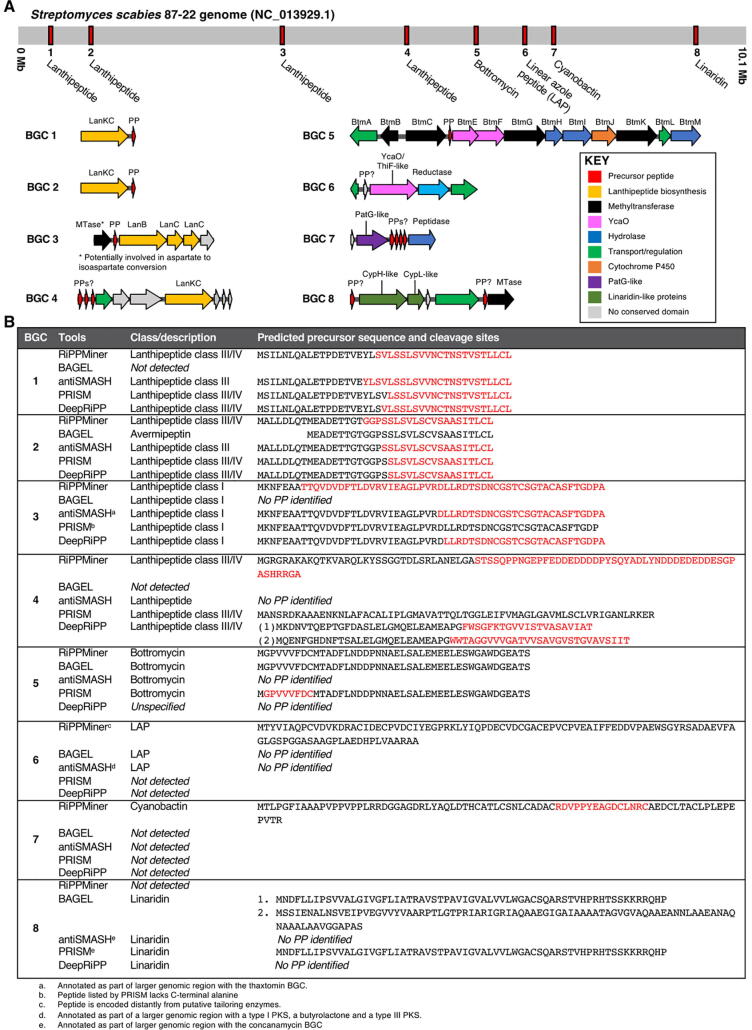
Overview of RiPP mining results for *Streptomyces scabies* 87–22. A. Genetic details of all RiPP BGCs identified by one or more tools. B. Summary of predictions made by each tool for a given RiPP BGC. Regions highlighted in red relate to predicted core peptides. (For interpretation of the references to colour in this figure legend, the reader is referred to the web version of this article.)

**Fig. 5:**
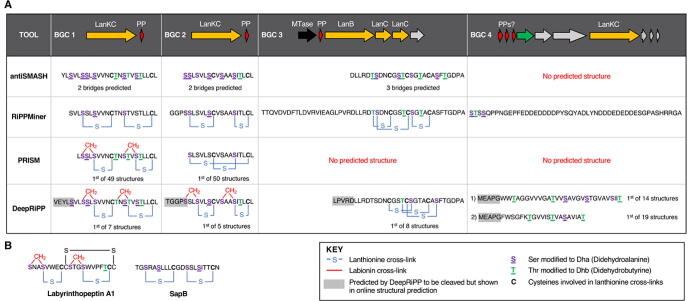
Summary of structural predictions provided for lanthipeptide BGCs by antiSMASH, RiPPMiner, PRISM and DeepRiPP. A. Summary of predictions (note: both PRISM and DeepRiPP predict multiple possible RiPP products and only the first prediction is visualised here). B. Structures of two characterised lanthipeptides whose BGCs have homology to BGC1 and BGC2.

In order to compare the BGC and PP recognition power of different RiPP mining tools that analyse a single whole genome as the input, we used the high-quality genome sequence of *Streptomyces scabies* 87.22 (NC_013929.1) as input for antiSMASH 5, BAGEL4, RiPPMiner, PRISM4 and DeepRiPP, and then carried out a detailed analysis of the outputs (Fig. 4, Fig. 5). We chose this organism as it is known to produce the RiPP bottromycin [20] and has multiple uncharacterised RiPP BGCs. In total, eight distinct RiPP BGCs were identified by the five tools (Fig. 4A). Surprisingly, only three of the eight BGCs were identified by all tools: a class III/IV lanthipeptide (BGC2), a class I lanthipeptide (BGC3) and bottromycin (BGC5).

DeepRiPP was the only tool that did not describe the class of the bottromycin BGC, whereas PRISM was the only tool that was able to identify both the correct core peptide and post-translational modifications for this known RiPP. Similarly, BAGEL did not identify two likely lanthipeptide BGCs that were identified by all other tools (Fig. 4B). The diversity of outputs extended to whether a precursor peptide was identified, where the cleavage site was predicted to be and what post-translational modifications are made to the core peptide (Fig. 4B and Fig. 5A). In the case of lanthipeptides, most tools predict a cleavage site, dehydrated residues and some predict final cyclised structures. For example, antiSMASH predicts cleavage sites, dehydrations for all Ser and Thr residues in a predicted core peptide, and states a predicted number of cross-links (Fig. 5A). It also provides a list of alternative masses based on fewer dehydrations. In contrast, RiPPMiner, PRISM and DeepRiPP provide full structural predictions (Fig. 5A). Despite similarity to characterised lanthipeptide BGCs, there was no consensus for predicted cleavage sites for any lanthipeptide, and the predicted sites and types of post-translational modifications also differed between tools.

The currently uncharacterised BGC1 provides a significant example of the challenges associated with RiPP predictions. RiPPMiner predicts its product will feature two lanthionine (Lan) residues [96], whereas both PRISM and DeepRiPP predict two labionin (Lab) residues, which feature a lanthionine thioether linkage and a carbacycle formed with an additional didehydroalanine or didehydrobutyrine [97]. PRISM and DeepRiPP themselves differ in the residues involved in these cyclisation reactions (Fig. 5A). Each of the seven structures proposed by DeepRiPP features the same cyclisation sites, whereas the top ten structures proposed by PRISM provide a number of alternative cyclisation sites, although all feature labionins.

Despite these differences, comparisons to known RiPPs highlights the difficulties in making robust RiPP predictions. This BGC is highly similar to a series of characterised lanthipeptide BGCs, including SapB [98] (*S. coelicolor*), avermipeptin [99] (*Streptomyces avermitilis*) and the labyrinthopeptins [97] (*Actinomadura namibiensis*) (Fig. 5B). However, despite similarities on a sequence level, SapB contains two Lan residues, avermipeptin contains 1 Lab and 1 Lan, and the labyrinthopeptins contain two Lab residues. Furthermore, RiPPquest-guided discovery of further members of this family, the informatipeptins [82], revealed that numerous derivatives were produced that differed at the *N*-terminus of the core peptide, highlighting the challenge of even identifying a true cleavage site. Therefore, RiPPMiner, PRISM and DeepRiPP all provide valid predictions for this BGC, especially as the Lan and Lab modifications cannot be distinguished by mass if a Lan modification is accompanied by an additional dehydration (as seen in SapB and the RiPPMiner BGC1 prediction). It is worth noting that BGC2 also has homology to these characterised BGCs (Fig. 5A). This highlights why predictive software should highlight these ambiguities when possible, and ideally provide users with a series of alternative predictions. This is carried out by DeepRiPP and PRISM (and partially by antiSMASH), although this could extend to proposing a small number of different cleavage sites, depending on the BGC. If this is not technically possible, genome mining tools should clearly highlight the limitations of their predictive powers when the user receives results.

The varied BGC and PP predictions provided by different RiPP mining tools highlights why it can be beneficial to use a variety of tools when analysing a genome, especially when some tools are better suited to certain RiPP subclasses. For example, the *S. scabies* “linaridin” BGC is identified by all tools apart from RiPPMiner, whereas RiPPMiner is the only tool to identify a “cyanobactin”-like BGC. The linaridin BGC is likely a genuine RiPP BGC, and was first bioinformatically identified along with the report of the first linaridin, cypemycin [100]. The cyanobactin-like BGC encodes a protein (SCAB_66631) with homology to PatG, which functions as an oxidase and macrocyclase in patellamide biosynthesis [101], [102]. However, further analysis shows that this homology is only to a domain of unknown function at the C-terminus of PatG [103]. Nevertheless, it is perhaps notable that SCAB_66631 is encoded alongside four short peptides as well as other putative biosynthetic proteins (Fig. 4A). This highlights the challenge of accurately interpreting some genome mining outputs.

The application of different tools can also be a useful way to provide confidence in a BGC prediction. PP sequences are not always predicted for identified BGCs, which highlights the importance of tools such as RiPPER and RODEO that can help identify PPs near a given RTE. On the other hand, more than one putative PP sequence was identified for some BGCs, and tools such as BAGEL and RiPPMiner also provide a list of other small ORFs nearby. In these cases, tools such as NeuRiPP may be useful to distinguish genuine RiPP PPs from other small peptides. Tools that integrate genomics and mass spectrometry, such as MetaMiner and DeepRiPP, are likely to prove increasingly useful in connecting sequence data to experimental data, although challenges remain in accurately predicting post-translational modifications, as well as the resulting masses and MS/MS fragmentation patterns.

## 7 Summary and outlook

The recent development and application of specialised RiPP genome mining tools has helped to uncover a vast landscape of RiPPs present in nature that were previously overlooked. This growing RiPP knowledgebase has led to advances in the algorithms used by these tools, which in turn is improving the systematic identification and annotation of RiPP BGCs in genomic data. This is also reflected by the development of multiple RiPP databases. As well as improved genomic analyses, several tools have also integrated the analysis of metabolomic data, searching for characteristic peptide residues and fragmentation patterns indicative of RiPP molecules. This has also allowed the exploration of large MS datasets that are now publicly available via resources such as GNPS.

Despite developments in genome mining, several challenges still remain in the field of RiPP discovery. Firstly, the identification of truly novel RiPP classes is a limitation of many current genome mining tools. This is partly due to the inherent nature of algorithms that rely on already known compounds. Furthermore, although genome mining facilitates the rapid identification of BGCs from genomic data, a bottleneck still remains with expressing and manipulating pathways in order to identify target molecules. Another drawback of genome mining is that, unlike activity-guided discovery, there is no guarantee that identified BGCs will produce a compound with clear biological activity. It is difficult to predict or prioritise BGCs that might encode NPs with a particular bioactivity of interest, given that self-resistance genes can be difficult to identify, if they are required at all. Therefore, extensive activity assays might be needed to determine the biological function of newly identified RiPPs. This is a challenge that is relevant across NPs.

However, the future of RiPP discovery holds a lot of exciting promise. The tools described in this review have been used to identify thousands of previously uncharacterised RiPP BGCs from a range of environments, and have also led to the isolation of structurally novel RiPPs with important antimicrobial bioactivity [36], [66]. Furthermore, an increased understanding of RiPP biosynthesis can enable the engineered production of unnatural peptides. For example, the characterisation of the highly promiscuous cyanobacterial lanthipeptide synthetase ProcM [104] led to the engineered production of a library of over 1 million cyclic peptides, including a potent inhibit of a protein–protein interaction critical for HIV infection [105]. Looking forward, we envisage that the use of exploratory tools, such as NeuRiPP and RiPPER (along with fully bespoke methods), will guide the discovery new RiPP families. The RODEO and RIPPER approach of using a user-defined protein of interest as “bait” differs from many other tools and provides greater flexibility due to the lack of strict rules for BGC detection. This is also better suited to the pan-genome analysis of related BGCs. Newly discovered BGC families can then be used to define conserved domains that, once incorporated into tools such as antiSMASH, DeepRiPP and BAGEL, will enable widespread discovery and understanding of related pathways. It is also clear that traditional activity-led screens can also be important for the discovery of new RiPP families, such as the recent discovery of darobactin (Fig. 2B), the founding member of a new RiPP class that selectively kills Gram-negative pathogens [106]. Discoveries such as this will also improve the bioinformatic rules used to identify RiPP BGCs.

## Declaration of Competing Interest

The authors declare that they have no known competing financial interests or personal relationships that could have appeared to influence the work reported in this paper.
